# Boosting the photocatalytic H_2_ evolution activity of type-II g-GaN/Sc_2_CO_2_ van der Waals heterostructure using applied biaxial strain and external electric field

**DOI:** 10.1039/d2ra00419d

**Published:** 2022-03-04

**Authors:** Francis Opoku, Samuel Osei-Bonsu Oppong, Albert Aniagyei, Osei Akoto, Anthony Apeke Adimado

**Affiliations:** Department of Chemistry, Kwame Nkrumah University of Science and Technology Kumasi Ghana ofrancis2010@gmail.com francisopoku@knust.edu.gh; Marine Engineering Department, Regional Maritime University P.O. Box GP 1115 Accra Ghana; Department of Basic Sciences, University of Health and Allied Sciences Ho Ghana

## Abstract

Two-dimensional (2D) van der Waals (vdW) heterostructures are a new class of materials with highly tunable bandgap transition type, bandgap energy and band alignment. Herein, we have designed a novel 2D g-GaN/Sc_2_CO_2_ heterostructure as a potential solar-driven photocatalyst for the water splitting process and investigate its catalytic stability, interfacial interactions, and optical and electronic properties, as well as the effects of applying an electric field and biaxial strain using first-principles calculation. The calculated lattice mismatch and binding energy showed that g-GaN and Sc_2_CO_2_ are in contact and may form a stable vdW heterostructure. *Ab initio* molecular dynamics and phonon dispersion simulations show thermal and dynamic stability. g-GaN/Sc_2_CO_2_ has an indirect bandgap energy with appropriate type-II band alignment relative to the water redox potentials. Meanwhile, the interfacial charge transfer from g-GaN to Sc_2_CO_2_ can effectively separate electron–hole pairs. Moreover, a potential drop of 3.78 eV is observed across the interface, inducing a built-in electric field pointing from g-GaN to Sc_2_CO_2_. The heterostructure shows improved visible-light optical absorption compared to the isolated g-GaN and Sc_2_CO_2_ monolayers. Our study demonstrates that tunable electronic and structural properties can be realised in the g-GaN/Sc_2_CO_2_ heterostructure by varying the electric field and biaxial strain. In particular, the compressive strain and negative electric field are more effective for promoting hydrogen production performance. Since it is challenging to tune the electric field and biaxial strain experimentally, our research provides strategies to boost the performance of MXene-based heterojunction photocatalysts in solar harvesting and optoelectronic devices.

## Abstract 2

Type-II g-GaN/Sc_2_CO_2_ van der Waals heterostructure with electronic properties has potential for nanoelectronics, optoelectronics and photovoltaic device applications.

## Graphical abstract

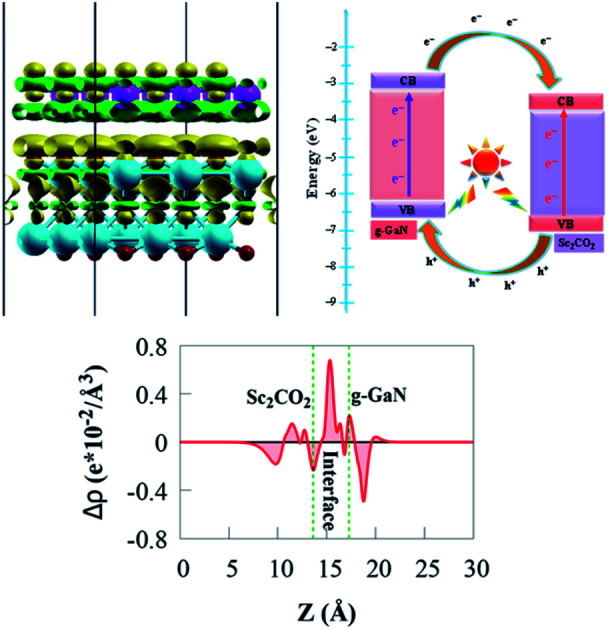

## I. Introduction

There are increased power and energy demands in applications, including industrial and household energy management, hybrid electric vehicles and cordless electric tools.^1^ To date, new approaches have been designed to generate alternative energy sources owing to the eventual depletion of fossil fuel.^2^ Photocatalysis has been an effective technology for producing sustainable and storable fuels using solar energy.^3^ Water is broadly regarded as the only accessible electron and proton source for fuel synthesis reactions, and this makes the hydrogen evolution reaction (HER) a critical component of solar energy technology.^4^ Due to the high energy efficiency and the practical and economic advantages, producing H_2_*via* the HER to replace the depleted fossil fuel is regarded as the most promising alternative clean energy carrier.^5^ Nonetheless, the low abundance and high cost of Pt-based H_2_ generation materials substantially restrict their use as an effective HER photocatalyst. Therefore, there is a higher challenge in developing a highly active, lower cost and more abundant noble metal free HER catalyst materials.^6^ Semiconductor-based photocatalyst materials, particularly those with strong structure stability and enhanced photoactivity, are promising solar energy conversion and storage materials.^7^

Lately, several two-dimensional (2D) have received much interest as potential materials for next-generation nanoelectronic, optoelectronic and solar conversion devices owing to their unique structures and outstanding properties.^8–11^ MXene with a hexagonal lattice has attracted much attention due to its promising applications in electronic devices.^12^ MXenes, such as 2D transition-metal carbonitrides, nitrides and carbides, have been prepared by isolating elementary MX layers from the bulk MAX materials,^13^ where M is an early transition metal, A is a group 13 or 14 element and X represents N or C element.^14^ MXenes have been studied as a possible energy storage solution for supercapacitors, CO_2_ conversion, photocatalytic hydrogen evolution and battery anodes in recent years.^15–18^ Most bulk MXenes show metallic features without bandgap energy, limiting their potential application.^13^ Nevertheless, they can tune from a metallic to a semiconducting state by surface functionalisation with groups, such as OH, F and O. For example, Sc_2_CO_2_, Sc_2_C(OH)_2_, Sc_2_CF_2_, Zr_2_CO_2_, Hf_2_CO_2_ and Ti_2_CO_2_ monolayers were predicted to be semiconductor materials.^19^ Materials with oxygen functional groups, such as Hf_2_CO_2_, Sc_2_CO_2_, Zr_2_CO_2_ and Ti_2_CO_2_, are typically the most stable and have excellent semiconductor properties.^20,21^ Particularly, Sc_2_CF_2_ and Sc_2_CO_2_ monolayers are demonstrated to have visible light optical absorption and high carrier mobilities.^22^ Sc_2_CO_2_, on the other hand, is distinguished from other semiconductor MXenes by its asymmetric oxygen-terminated structure.^23^ Because of its nonvanishing electric dipole moment,^24^ Sc_2_CO_2_ is a good material when investigating the effect of polarity on heterostructure band alignment. Because it has a stable structure with an indirect bandgap and can resist high temperature,^25^ graphene-like gallium nitride (g-GaN) is one of the next generation 2D materials.^26,27^ Recently, g-GaN was synthesised using the migration-enhanced encapsulated growth technique,^28^ and its electronic properties may be easily controlled using a stacking effect or an external electric field.^29^

Surprisingly, recent theoretical studies^30–32^ suggested that several 2D-based materials may be suitable for water splitting applications. Nonetheless, because they are atomically thin, photogenerated charge carriers have a short lifetime,^30,33^ limiting their practical applicability. As a result, employing 2D monolayer as photocatalyst for water splitting is difficult and complicated. 2D van der Waals (vdW) heterostructures, which combine the characteristics of their individual components, are now regarded as a potential approach of fabricating nanoelectronic, optoelectronic and solar energy conversion devices.^34,35^ In the vdW heterostructure, band alignments are classified as type-I, type-II, or type-III, with each having a distinct application that allows for the fabrication of various electronic devices.^36^ Many newly designed 2D-based vdW heterostructures^37,38^ have type-II band alignment with a reduced recombination rate of photogenerated charge carriers with the help of the valence-band offset (VBO) and conduction-band offset (CBO),^38^ extending photogenerated charge carrier lifetimes and enhancing water splitting efficiency.

More crucially, g-GaN sharing nearly identical lattice constants and a similar hexagonal crystal structure with Sc_2_CO_2_ is beneficial to the fabrication of g-GaN/Sc_2_CO_2_ heterostructure, which are highly expected and of great interest.^20,39^ Therefore, we systematically investigate the underlying mechanism of charge transfer, interface interaction, electronic structures, relative stability, band alignments and optical absorption properties of g-GaN/Sc_2_CO_2_ heterostructure as a possible water splitting photocatalyst using density functional theory (DFT) calculations. Considering that g-GaN and Sc_2_CO_2_ monolayers have been reported to show outstanding electronic properties under strain,^29^ the effect of biaxial strain and electric field on the structural and electronic (bandgaps and band edge positions) properties of g-GaN/Sc_2_CO_2_ heterostructure was also investigated. Our findings should open the way for a new generation of optoelectronic devices and develop vdW heterostructure as viable candidates for photocatalytic application.

## II. Computational details

First-principles calculations were carried out within the plane-wave DFT framework, as implemented in the Quantum Espresso package,^40^ with optimised norm-conserving Vanderbilt pseudopotentials.^41^ For the exchange–correlation energy, structural optimisations were done using the generalised gradient approximation (GGA) with the Perdew–Burke–Ernzerhof (PBE) functional.^42^ For wavefunctions and charge density, the plane wave kinetic energy cutoffs were set to 80 and 520 Ry, respectively. The interlayer vdW interactions and dipole were treated using the dispersion corrections method of Grimme (DFT-D3(BJ)).^43^ To minimise spurious interaction between periodic pictures, a vacuum spacing of 30 Å was used. By fully relaxing both ionic locations and cell vectors until the maximum residual force and energy were less than 10^−3^ Ry per bohr and 10^−8^ Ry, respectively, optimal lattice parameters were achieved. The Brillouin zone (BZ) for g-GaN, Sc_2_CO_2_ and g-GaN/Sc_2_CO_2_ heterostructure was sampled using a Monkhorst–Pack^44^ with *k*-points of 14 × 14 × 1, 12 × 12 × 1, and 12 × 12 × 1, respectively. To retain the same number of *k*-points within the irreducible BZ for all structural models, no symmetry was used in all computations. The Methfessel–Paxton smearing method was used with a broadening of 0.002 Ry along the high symmetry zone. Because the PBE functional underestimates the bandgap energy of semiconductors, the Heyd–Scuseria–Ernzerhof (HSE06) hybrid functional (mixing parameter 0.25, screening parameter 0.2 Å^−1^)^45^ was used. HSE06 has been shown to be a dependable functional for calculating optical and electronic properties.^46^ To analyse the kinetic stability of the heterostructure and monolayers, phonon dispersion calculations were done using the density functional perturbation theory^47^ within the Phonopy package^48^ as implemented in the Quantum ESPRESSO code. The real-space force constants were computed using the finite displacement technique from the Hellmann–Feynman forces by introducing displacements to supercells.^49^ The force constants were then used to calculate the dynamical matrices and phonon frequencies. Besides, the thermal stability was evaluated using *ab initio* molecular dynamics (AIMD) simulations with the Nosé–Hoover scheme^50,51^ at an ambient temperature of 300 K for 70 ps within each 1 fs time step. To consider the constraints of lattice translational, we design 4 × 4 supercells of Sc_2_CO_2_/g-GaN heterostructure for the AIMD simulations. Visualisations of charge density difference, electron localisation function and optimised structures were obtained with the XCrySDen package.^52^

## III. Results and discussion

**Fig. 1:**
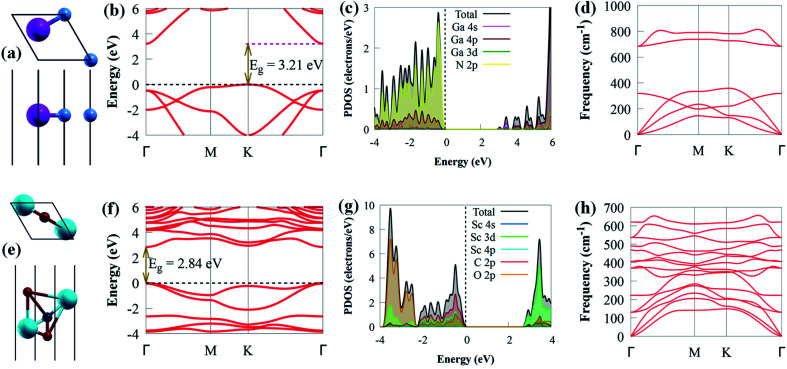
(a) The geometrical top view (above), side view (below), (b) band structure, (c) PDOS and (d) phonon of g-GaN monolayer. (e) Top view (above), side view (below), (f) band structure, (g) PDOS and (h) phonon of Sc_2_CO_2_ monolayer. Magenta, blue, cyan, red and grey spheres represent Ga, N, Sc, O and C atoms, respectively. The Fermi level is set to zero for clarity.

**Fig. 2:**
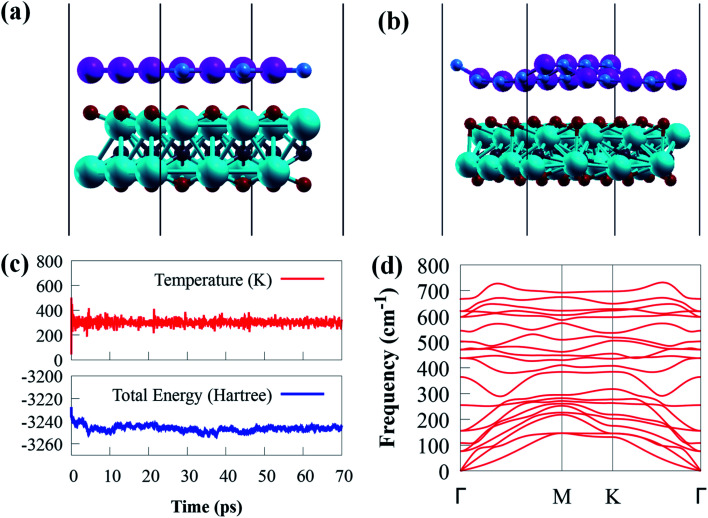
(a) Schematic illustration of the side view of g-GaN/Sc_2_CO_2_ heterostructure. (b) Snap shot of AIMD simulations g-GaN/Sc_2_CO_2_ heterostructure after 70 ps. (c) The total energy and temperature fluctuations during the AIMD simulations under the temperature of 300 K after 70 ps and (d) phonon dispersion curve for the g-GaN/Sc_2_CO_2_ heterostructure.

Before delving into the optical and electronic properties of g-GaN/Sc_2_CO_2_ heterostructure, we first examine the lattice constants of g-GaN and Sc_2_CO_2_ monolayers, which are optimised as 3.438 and 3.253 Å, respectively. Our optimised lattice parameters agreed with earlier results,^8,39^ signifying the dependability of our computational method. The relaxed crystal structures, projected density of states (PDOS), electronic band structures and phonon of g-GaN and Sc_2_CO_2_ monolayers are depicted in Fig. 1. The indirect bandgap of g-GaN monolayer is 3.21 eV. The conduction band minimum (CBM) and valence band maximum (VBM) of g-GaN monolayer are positioned at the *Γ* and *K* points, respectively. However, the direct bandgap in Sc_2_CO_2_ monolayer (2.84 eV) is situated at *Γ* point of the BZ. These findings are consistent with previously reported values.^8,53^ The PDOS of g-GaN monolayer showed that the VBM mainly comprises N 2p states, whereas the CBM is dominated by Ga 4s state (Fig. 1c). The PDOS results agreed with earlier studies.^54^ Analysis of PDOS further reveals that the CBM of Sc_2_CO_2_ monolayer is dominated by the Sc 3d state, while C 2p states mainly contribute to the VBM (Fig. 1g). The PDOS results of Sc_2_CO_2_ are in agreement with earlier study.^53^

g-GaN/Sc_2_CO_2_ heterostructure was designed from the unit cell of g-GaN and Sc_2_CO_2_ monolayers, as shown in Fig. 2a.

Generally, a stable heterostructure may favourably synthesise when the lattice mismatch is 5%.^55^ The interlayer lattice mismatch between g-GaN and Sc_2_CO_2_ monolayers is 5.38%, which is acceptable and accessible in the experimental synthesis because their interfaces are flexible and can accommodate this mismatch.^53^ Thus, the small lattice mismatch within 5.38% in all directions means high possibility to grow heterostructure among these compounds. For example, both experimental and theoretical studies on CsPbI_3_/GaN heterostructure confirm that the small lattice mismatch (2.1%) contributes to the in-plane growth of CsPbI_3_ on the mica substrate.^56^ Moreover, the lattice mismatch between MoS_2_ and Nb_2_CO_2_ monolayers is only about 1.5%, which indicates that the fabricated heterostructure is desirable. Expectedly, MoS_2_/Nb_2_CO_2_ heterostructure has been synthesised through a facile hydrothermal chemical method.^57^ Also, a lattice mismatch of less than 0.16% offers the experimental synthesis of PtSe_2_/GaN heterostructure. Zhuo *et al.*^58^ have successfully synthesised the PtSe_2_/GaN vdW heterostructure experimentally that is used for self-powered deep ultraviolet photodetector. In the supercell, the positive sign reflects a small lattice constant of the g-GaN monolayer with respect to the Sc_2_CO_2_ monolayer. The interface binding energy (*E*_b_) of g-GaN/Sc_2_CO_2_ heterostructure is computed using the following equation to analyse the structural stability as follows:^59^

1 *E*_b_ = *E*_g-GaN/Sc_2_CO_2__ − *E*_g-GaN_ − *E*_Sc_2_CO_2__/*S*,

where *E*_Sc_2_CO_2_/g-GaN_, *E*_g-GaN_ and *E*_Sc_2_CO_2__ are the total energies of g-GaN/Sc_2_CO_2_ heterostructure, g-GaN and Sc_2_CO_2_ monolayers, respectively. *S* denote the surface area. The calculated binding energy of −37.08 meV Å^−2^ suggests that g-GaN/Sc_2_CO_2_ heterostructure is thermodynamically stable. The results show that the *E*_b_ of g-GaN/Sc_2_CO_2_ vdW heterostructure is much lower than that of other vdW heterostructures, such as MoS_2_/GaN (−24.14 meV Å^−2^),^60^ PtSe_2_/GaN (−6.41 meV Å^−2^),^61^ Zr_2_CO_2_/MoS_2_ (−10.42 meV Å^−2^),^62^ g-GaN/(MoS_2_) WS_2_ (−19.52 (−19.61) meV Å^−2^),^8^ Sc_2_CO_2_/h-BN (28 meV Å^−2^),^63^ g-GaN/(MoSe_2_) WSe_2_ (−21.46 (−21.87) meV Å^−2^)^8^ and GaN/BAs (−8.56 meV Å^−2^),^64^ indicating that the g-GaN/Sc_2_CO_2_ heterostructure possess higher stability. This value is also more negative than the typical vdW binding energy (−13 to −20 meV Å^−2^),^65^ indicating that weak vdW interaction exists between g-GaN and Sc_2_CO_2_ monolayers. Furthermore, the interlayer distances of 3.206 Å are close to 3.336 Å observed in a typical vdW 2D material, such as graphite,^66^ suggesting that g-GaN/Sc_2_CO_2_ heterostructure is generated by vdW forces.

In addition, the change in total energy and temperature during the AIMD simulation is depicted in Fig. 2b and c. The results show that no visible bond breaking occurs after 70 ps at room temperature, and the overall energy and temperature variations are within a limited range, showing the thermal stability of g-GaN/Sc_2_CO_2_ heterostructure. This also confirmed that experimental fabrication is highly expected.^67,68^

The phonon dispersion spectrum is a trustworthy method for determining the stability of a structure.^69^ For all modes along the high symmetry BZ direction, a dynamically stable structure has no imaginary phonon frequencies. The phonon dispersion spectra of g-GaN/Sc_2_CO_2_ heterostructure, as well as g-GaN and Sc_2_CO_2_ monolayers, are calculated to analyse their kinetic stabilities. The phonon spectrum of Sc_2_CO_2_ (g-GaN) monolayer in Fig. 1d and h consist of 15 (6) branches, including 3 (3) acoustic and 12 (3) optical modes composed of five (two) atoms in the unit cell. The out-of-plane acoustic (ZA), transversal acoustic (TA) and longitudinal (LA) branches are linear near the *Γ* point of the BZ. It is worth noting that all the frequencies of g-GaN/Sc_2_CO_2_ heterostructure (Fig. 2d), g-GaN and Sc_2_CO_2_ monolayers are positive, confirming the dynamic stability.

**Fig. 3:**
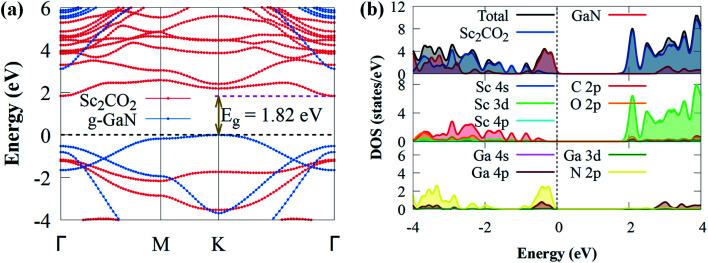
(a) The projected electronic band structure of g-GaN/Sc_2_CO_2_ heterostructure; the dotted horizontal line signifies the Fermi energy level. (b) The local density of states of Sc, O, C, Ga and N atoms of Sc_2_CO_2_ and g-GaN in the heterostructure. The dash vertical or horizontal line represents the Fermi level.

Having established the dynamic and thermal stability, we then investigated the projected band structure of g-GaN/Sc_2_CO_2_ heterostructure, as given in Fig. 3a.

When a g-GaN/Sc_2_CO_2_ heterostructure is built, the band structures of both the Sc_2_CO_2_ and g-GaN monolayers are relatively well conserved. Compared to the direct bandgap of Sc_2_CO_2_ monolayer, the g-GaN/Sc_2_CO_2_ heterostructure has an indirect bandgap (1.82 eV) with VBM and CBM positioned at the *K* and *Γ* points of the BZ, respectively. The indirect bandgap semiconductor corresponds with other vdW heterostructures.^26,70,71^ The bandgap energy was lower than g-GaN/BSe (2.268 eV),^72^ MoSSe/g-GaN (2 eV),^73^ GaN/SiS (2.45 eV),^74^ WSSe/g-GaN (2.14),^73^ GeC/GaN (2.76 eV)^75^ and BlueP/Sc_2_CO_2_ (1.91 eV)^76^ vdW heterostructures. The smaller bandgap may be more conducive to the transfer and separation of photogenerated charge carriers, highlighting their potential application as visible light photocatalysts for H_2_ generation compared to the above vdW heterostructures. Furthermore, the heterostructure exhibited a lower bandgap than the g-GaN and Sc_2_CO_2_ monolayers, implying that electron excitation from the VBM to the CBM is simpler when the heterostructure is exposed to visible light. Furthermore, it had a much higher bandgap than the water splitting redox potential energy (1.23 eV), showing that the electronic structure of g-GaN/Sc_2_CO_2_ heterostructure makes it a visible light photocatalyst.^74^ The projected DOS and band structure shows that the Sc_2_CO_2_ monolayer dominates the CBM, whereas the g-GaN monolayer dominates the VBM, indicating that the VBM and CBM states are spatially separated. As a result, at the interface, an inherent type-II (staggered) band alignment is formed, which could aid in the effective separation of photoexcited electron–hole pairs once the vdW heterostructure is illuminated by sunlight. Also, because staggered type II band alignment heterostructures allow larger offsets on one side, substantial carrier confinement is always present.^36^ As a result, the heterostructure has potential applications in light-emitting diodes, lasers and solar energy conversion devices.^77^ The PDOS was computed and shown in Fig. 3b to help comprehend the band alignment. The VBM is predominantly derived from the N 2p orbitals, while the Sc 3d orbitals contribute to CBM. This validates the type-II band alignment, which effectively separates electron–hole pairs in both layers.^78^

**Fig. 4:**
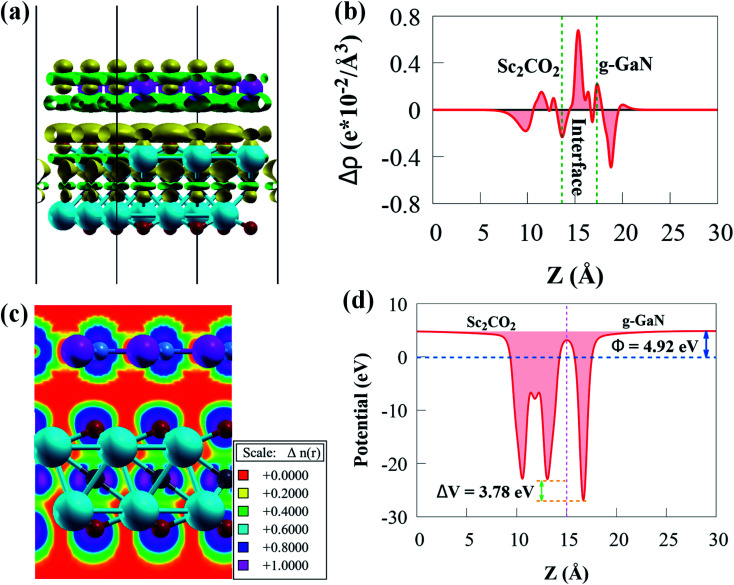
(a) Side view of the charge density difference (CDD) with an isovalue of 0.0005 e bohr^−3^. (b) The plane-averaged CDD (c) electron localisation function and (d) the plane-averaged electrostatic potential perpendicular to g-GaN/Sc_2_CO_2_ heterostructure.

As mentioned above, the g-GaN/Sc_2_CO_2_ heterostructure exhibit type-II band alignment with promising application in photocatalytic water splitting. The built-in electric field has a vital influence on the lifetime of photogenerated charge carriers, which is well recognised. As a result, we evaluate the interfacial properties of g-GaN/Sc_2_CO_2_ heterostructures to check if a built-in electric field exists by analysing the charge transfer, as illustrated in Fig. 4a.

The green and yellow colours represent charge depletion and accumulation, respectively. Because of the vdW interaction at the interface, charge transfer is redistributed at the interface of g-GaN/Sc_2_CO_2_ heterostructure. The g-GaN monolayer donates electrons, whereas Sc_2_CO_2_ monolayer receives electrons. Thus, charge redistributions indicate interfacial charge transfer from g-GaN to Sc_2_CO_2_, which induces a built-in electric field pointing from g-GaN to Sc_2_CO_2_. The built-in electric field can cause photogenerated electrons and holes to separate spatially on various monolayers. According to the Löwdin charge population analysis,^79^ about 0.033 e per unit cell are transported from the g-GaN monolayer to the Sc_2_CO_2_ monolayer, resulting in a weak built-in electric field pointing from g-GaN monolayer to Sc_2_CO_2_ monolayer. The weak interaction between g-GaN and Sc_2_CO_2_ monolayers is indicated by such a small charge transfer. Even though this built-in electric field is small, we cannot overlook its vital significance in preventing charge carrier recombination rate, which can play an important influence in enhancing carrier mobility and prolonging their lifetime.

The Δ*ρ*(*z*) in Fig. 4b is negative and positive at the interface near g-GaN and Sc_2_CO_2_ monolayers, respectively. This confirms that electrons are transferred from g-GaN monolayer to Sc_2_CO_2_ monolayer through the vdW gap, which is primarily responsible for the band offset. Because the average electrostatic potential of g-GaN monolayer is lower than that of Sc_2_CO_2_ monolayer, as shown in Fig. 4c, a potential drop of 4.18 eV is observed across the interface, generating a built-in electric field. The potential drop correlates to a strong electrostatic field across the interface, which may have a significant impact on carrier dynamics and charge transfer if the g-GaN monolayer is used as an electrode. As a result, a strong driving force between the two monolayers is produced and electrons may be propelled across the interface from g-GaN monolayer to Sc_2_CO_2_ monolayer. The band offset is principally caused by the significant potential drop, which will aid in the separation and transfer of electron–hole pairs.^80^

The electron localisation function (ELF) may also be used to visualise the weak interfacial contact in the g-GaN/Sc_2_CO_2_ heterostructure. The spatial localisation extent of reference electrons is described by ELF, which is a continuous variable with values ranging from 0 to 1.^81^ ELF of 0 denotes total electron decentralisation, whereas ELF of 1 represents complete electron localisation.^81^ As seen in Fig. 4d, no electron is localised in the interface area, confirming the existence of vdW force.

The charge density distribution at the heterostructure interface can influence the band alignment of semiconductor photocatalyst materials. The work function (*Φ*) of a photocatalyst material is an important electronic property that provides an in-depth knowledge of charge transfer and the relative location of the Fermi energy level.^82^*Φ* is affected by the vacuum energy (*E*_vacuum_) and the Fermi energy (*E*_F_) as follows:

2 *Φ* = *E*_vacuum_ − *E*_Fermi_

**Fig. 5:**
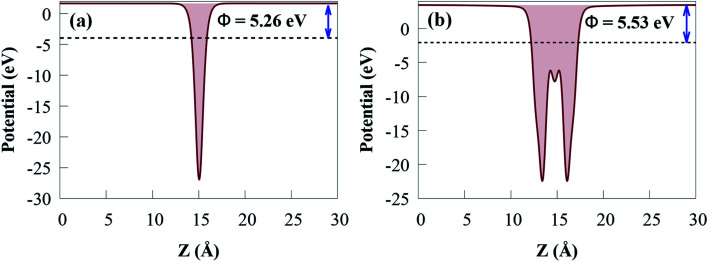
Calculated work function of (a) g-GaN and (b) Sc_2_CO_2_ monolayers.

For g-GaN and Sc_2_CO_2_ monolayers shown in Fig. 5, the work functions are 5.26 and 5.53 eV, respectively, which agreed with previous reported values.^53,83^

As Sc_2_CO_2_ coupled with g-GaN, the *Φ* difference between g-GaN and Sc_2_CO_2_ caused electrons in the g-GaN monolayer to spontaneously transfer to Sc_2_CO_2_ monolayer, and similarly, holes transfer to g-GaN, bringing the Fermi levels of the two monolayers to level up. Accordingly, electrostatics contribution makes the g-GaN and Sc_2_CO_2_ potentials positively and negatively charged, respectively, across the interfacial region. After the two monolayers have reached an equalised Fermi energy level, a built-in electric field pointing from g-GaN to Sc_2_CO_2_ will be established. Therefore, the electrons accumulated in Sc_2_CO_2_ surface cannot transfer back to g-GaN, thus limiting the direct recombination rate of charge carriers. Because of interface formation and charge transfer, the *Φ* of g-GaN/Sc_2_CO_2_ heterostructure (4.92 eV) in Fig. 4c is lower than that of the constituent monolayers.^73^ Because of the low *Φ*, light irradiation would make the electron transfer between the VBM and CBM edges easier.^76^

**Fig. 6:**
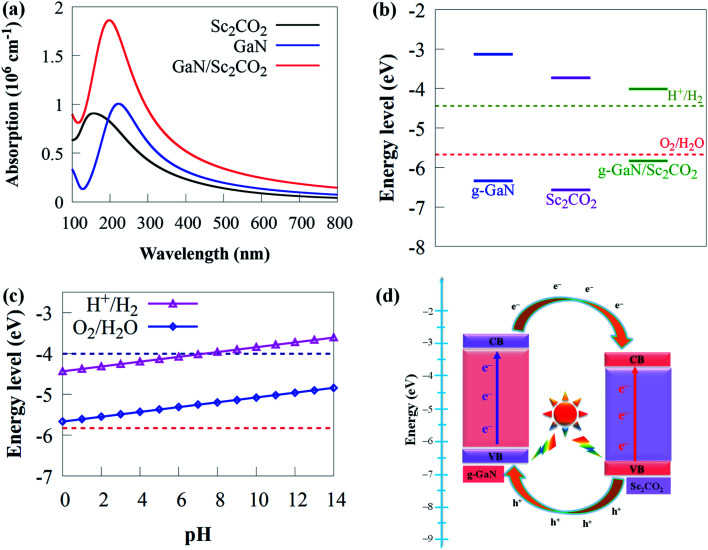
(a) The absorption spectra of g-GaN/Sc_2_CO_2_ heterostructure and the individual g-GaN and Sc_2_CO_2_ monolayers. (b) The band alignment of 2D g-GaN/Sc_2_CO_2_ heterostructure and individual g-GaN and Sc_2_CO_2_ monolayers. (c) The absolute energy bands for the CBM and VBM of g-GaN/Sc_2_CO_2_ heterostructure with respect to pH (0–14). (d) Schematic diagram showing charge carriers transfer at the g-GaN/Sc_2_CO_2_ interface.

Because it can catch visible light to trigger photocatalytic redox processes, the optimal bandgap for a semiconductor photocatalyst is ∼2 eV.^84,85^ To promote photoactivity, rational modulation of bandgap is vital for efficient use of visible light absorption. Hence, it is significant to tune the bandgap and the band potential to prolong the visible light response to a broader wavelength region. Therefore, the novel 2D g-GaN/Sc_2_CO_2_ photocatalyst material designed should have enough optical absorption properties to capture more solar energy. As a result, as shown in Fig. 6, comparisons of optical absorption spectra are carried out between the novel 2D g-GaN/Sc_2_CO_2_ heterostructure and individual g-GaN and Sc_2_CO_2_ monolayers.

Both g-GaN and Sc_2_CO_2_ monolayers exhibit a weak absorption edge and intensity. The visible light absorption edge and intensity of g-GaN/Sc_2_CO_2_ heterostructure is higher than that of the individual monolayers. Because it results in type-II band alignment and benefits the effective separation of charge carriers, interlayer coupling between g-GaN and Sc_2_CO_2_ monolayers may be responsible for the enhanced redshift of absorption, thereby improving the photocatalytic performance. As a result, g-GaN/Sc_2_CO_2_ heterostructure can function as high-performance solar energy absorbers, which is advantageous for achieving a much better photocatalyst than the monolayers.

The band edge of photocatalytic water splitting materials must be at a suitable position in addition to having ideal bandgap energy. The valence band (VB) and the conduction band (CB) edge potentials of g-GaN and Sc_2_CO_2_ monolayers are evaluated using the bandgap and the absolute electronegativity of the atoms as follows:^86^

3 *E*_VB_ = *χ* − *E*_e_ + 0.5*E*_g_

4 *E*_CB_ = *E*_VB_ − *E*_g_

where *E*_CB_ and *E*_VB_ are the CB and VB potential, respectively, *E*_g_ is the estimated bandgap, *χ* is the absolute electronegativity and *E*_e_ is the energy of free electrons (4.5 eV) on the hydrogen scale. The *χ* values of g-GaN and Sc_2_CO_2_ are calculated as 4.833 and 5.247 eV, respectively, using the method proposed by Pearson.^87^ The CBM of g-GaN, Sc_2_CO_2_ and g-GaN/Sc_2_CO_2_ are calculated as −3.13, −3.73 and −4.01 eV, respectively, while the respective VBM are −6.34, −6.57 and −5.83 eV, respectively. Because photocatalysis occurs in water, the pH of water influences it. Thus, acid–base characteristics are essential for the performance of photocatalyst materials. As a result, we also address the impacts of acid–base characteristics on the photoactivity of g-GaN/Sc_2_CO_2_ heterostructure by varying the pH values. The reduction and oxidation potentials relationship between water and pH is given by the Nernst equation^88,89^ as −4.44 eV + pH × 0.059 eV and −5.67 + pH × 0.059 eV, respectively. For water-splitting reactions, a photocatalyst must meet the requirement that the VBM must lie below the oxidation potential (*E*_O_2_/H_2_O_) of water (−5.67 eV) and the CBM must lie above the reduction potential (*E*_H^+^/H^2^_) of water (−4.44 eV) at pH of 0 and are −5.257 and −4.027 eV at pH of 7, respectively. The band alignment of g-GaN, Sc_2_CO_2_ and g-GaN/Sc_2_CO_2_ are given in Fig. 6b. The CBM and VBM of g-GaN, Sc_2_CO_2_ and g-GaN/Sc_2_CO_2_ are in photocatalytically favourable locations, indicating that these materials are appropriate for water splitting. According to previous research, the band edges of g-GaN and Sc_2_CO_2_ monolayers are appropriate for water splitting.^70,76^ Nevertheless, their bandgap energies are wide (>2.20 eV), restraining their capacity to absorb solar energy.^70^ The band edge potentials of g-GaN/Sc_2_CO_2_ heterostructure, on the other hand, is closer to the oxidation and reduction potentials than the band edges of g-GaN and Sc_2_CO_2_ monolayers. These potentials are strong enough to induce photogenerated holes and electrons to dissociate water into O_2_ and H_2_, respectively, showing that g-GaN/Sc_2_CO_2_ heterostructure is a better photocatalyst for water splitting at a pH of 0 than individual monolayers.

Increasing the pH value from 0 (strong acid) to 3 (relatively weak acid) and eventually to 7 (neutral) retains the CBM more positive than the reduction potential, showing that the g-GaN/Sc_2_CO_2_ heterostructure has a favourable response for photocatalytic water splitting (Fig. 6c). We predict that novel 2D g-GaN/Sc_2_CO_2_ heterostructure straddling the redox potential of water at pH of 0–7 are beneficial for water splitting for large-scale and cost-efficient hydrogen production. However, when the pH is raised to 8, the CBM is pushed lower than the reduction potential, indicating that the overall photocatalytic performance has decreased. Thus, given the overpotential factor, it may not have been adequate for oxidation or reduction in a strong acid or base environment. These findings suggest that water splitting processes benefit from a mild acid environment similar to GaTe/C_2_N^90^ and SiC (g-SiC)/MoS_2_ (ref. 91) vdW heterostructure.

The schematic illustration of charge transfer at g-GaN/Sc_2_CO_2_ interface is given in Fig. 6d. Arrows represent the transfer of photogenerated electron–hole pairs at the interface. When the g-GaN/Sc_2_CO_2_ heterostructure is irradiated by sunlight, photogenerated electron–hole pairs are generated by absorbing energy larger than the bandgap energy. Subsequently, electrons will transfer from the VB to the CB of the respective monolayers. Moreover, driven by the CBO of 0.6 eV, electrons staying in the CB of g-GaN monolayer tend to transfer to the CB of Sc_2_CO_2_ monolayer. Meanwhile, the photogenerated holes in the Sc_2_CO_2_ monolayer move to the VB of g-GaN monolayer, driven by VBO of 0.23 eV. Consequently, this process can promote charge separation, resulting in low electron–hole pair recombination rate and allowing water redox reactions to be efficiently performed on the respective components. We predicted that H_2_ reduction would come from Sc_2_CO_2_ monolayer, while O_2_ oxidation from g-GaN monolayer under sunlight irradiation.

**Fig. 7:**
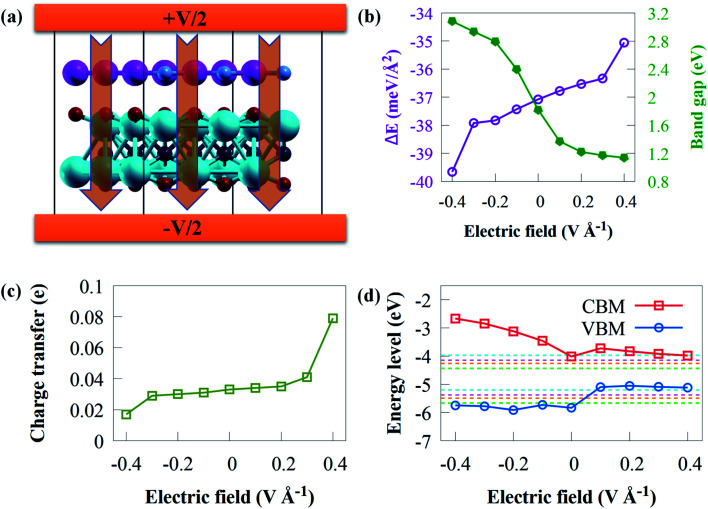
(a) Schematic illustration of applied external electric field perpendicularly to g-GaN/Sc_2_CO_2_ vdW heterostructure. Variation of the (a) binding energy, (b) bandgap energy, (c) charge transfer and (d) band edge positions of g-GaN/Sc_2_CO_2_ heterostructure against external electric field.

The use of an electric field is frequently simple theoretically, and it offers the advantages of reversibility and high operability since the nature of the system does not alter when electric field is withdrawn.^92^ Applying an external electric field is an effective technique of modifying the electronic property of vdWs heterostructure,^93^ which can be used in nanoelectronic devices. Recent theoretical studies^94^ have reported that the introduction of an electric field results in a tunable electronic property. Practically, materials are normally affected by an external electric field when used in electronic devices.^95^ Herein, the influence of an external electric field on the electronic and structural properties of g-GaN/Sc_2_CO_2_ vdW heterostructure are carefully studied since a tunable bandgap engineering is experimentally meaningful.^96^ Two opposite electric fields (+*z*, −*z*) vertical to the stacking layers were examined. A positive external electric field (+*z*) corresponds to the direction of the intrinsic built-in electric field in g-GaN/Sc_2_CO_2_ vdW heterostructure. In this study, the external negative electric field (−*z*) points from Sc_2_CO_2_ monolayer to the g-GaN monolayer, while the external positive electric field direction points from g-GaN monolayer to the Sc_2_CO_2_ monolayer. Herein an external electric field varying from −0.4 to +0.4 V Å^−1^ with steps of 0.1 V Å^−1^ is applied, as shown in Fig. 7a.

To gain fundamental insight into the influence of external electric field on the interfacial interaction, the *E*_b_ is shown in Fig. 7b. *E*_b_ remains negative within the entire external electric field range, demonstrating the thermodynamic stability of g-GaN/Sc_2_CO_2_ heterostructure under an external electric field.

The bandgap energy against applied external electric field is given in Fig. 7b. The bandgap energy is sensitive to the intensity and direction of external electric field. The results indicated that when the electric field strength increased, the bandgap energy decreased linearly, where +0.1 V Å^−1^ causes a steeply decrease in the bandgap energy. However, a gradual decrease is observed from +0.2 to +0.4 V Å^−1^, where the bandgap decreases from 1.33 eV with +0.2 V Å^−1^ to 1.14 eV with +0.4 V Å^−1^. This indicates that g-GaN/Sc_2_CO_2_ heterostructure retains its semiconducting character. The highly narrow bandgap induced by an external positive electric field might not be suitable for the overall water splitting, whereas a wide bandgap might reduce the performance to absorb solar energy. From +0.2 to +0.4 V Å^−1^, the bandgap energies varying from 1.33 to 1.14 eV are lower than the minimum bandgap energy (1.23 eV) essential for photocatalytic water splitting application. However, these bandgap energies fall within photovoltaic range (1.1 to 1.5 eV);^97,98^ hence, g-GaN/Sc_2_CO_2_ heterostructure may be good for photovoltaic cells with applied electric field of +0.2 to +0.4 V Å^−1^. Similar tunable bandgap energies under positive electric field in other heterostructures has also been reported.^99^ On the other hand, when a positive electric field is applied, the bandgap energy increase from 1.82 eV at 0 V Å^−1^ to 3.08 eV at −0.4 V Å^−1^. The results reveal that both the magnitude and the direction of the external electric field could be used to tune the bandgap energy of g-GaN/Sc_2_CO_2_ heterostructure. Based on the above discussion, g-GaN/Sc_2_CO_2_ heterostructure is a potential candidate for catalysis and optoelectronic devices.

Apart from the tunable bandgap, the applied electric field can considerably boost the electron redistributions at the interface and the interactions between the isolated monolayers.^94^ The charge transfer against electric field are calculated as given in Fig. 7c. The direction of external positive electric field can boost the transfer of electrons from g-GaN to Sc_2_CO_2_. When Δ*q* increases monotonically under external positive electric field, continuous charge transfer from g-GaN to Sc_2_CO_2_ occurs. This attenuated charge accumulation suggests that the increasing external negative electric field interferes with interfacial interactions. However, compared with the case of an external electric field of 0 V Å^−1^, the number of electrons transferring from g-GaN to Sc_2_CO_2_ decreases when the applied negative external electric field increases.

To offer a fundamental understanding of band edge potentials of g-GaN and Sc_2_CO_2_ monolayers in the heterostructure, we plotted the potentials against external electric field at pH of 0 to 7 (Fig. 7d). The CBM and VBM composed of Sc_2_CO_2_ and g-GaN monolayers move upward as the negative electric field increases, particularly the CBM band. The CBM and VBM potentials of g-GaN/Sc_2_CO_2_ heterostructure under an electric field of −0.1 to −0.4 V Å^−1^ are all well aligned to straddle the redox potentials of water, and this can practically be employed as photocatalytic water splitting at pH 0 to 7. However, g-GaN/Sc_2_CO_2_ heterostructure with positive external electric field exhibit a more negative potential with a higher reduction ability to generate H_2_*via* water splitting process, but the VBM could not straddle the water oxidation potential (*E*_O_2_/H_2_O_) of water.

Electronic structures of 2D materials can be tuned by mechanical strain.^100^ Compressive or tensile strain has been demonstrated in experiments by several techniques, such as lattice mismatch, bending, external load, *etc.*^101^ It is generally known that strain may change the band edge positions and band gaps of 2D photocatalytic materials and reduce the energy of hydrogen absorption.^102^ Here, we have systematically investigated the influence of strain on electronic and structural properties by applying a biaxial strain in the range of −6% (compressive) to +6% (tensile). By changing the lattice parameters, the in-plane biaxial strain, which is defined as *ε* = (*a*_1_ − *a*_0_)/*a*_0_ × 100% is simulated. Here *a*_0_ and *a*_1_ represent the lattice constant of unstrained and strained system, respectively.

**Fig. 8:**
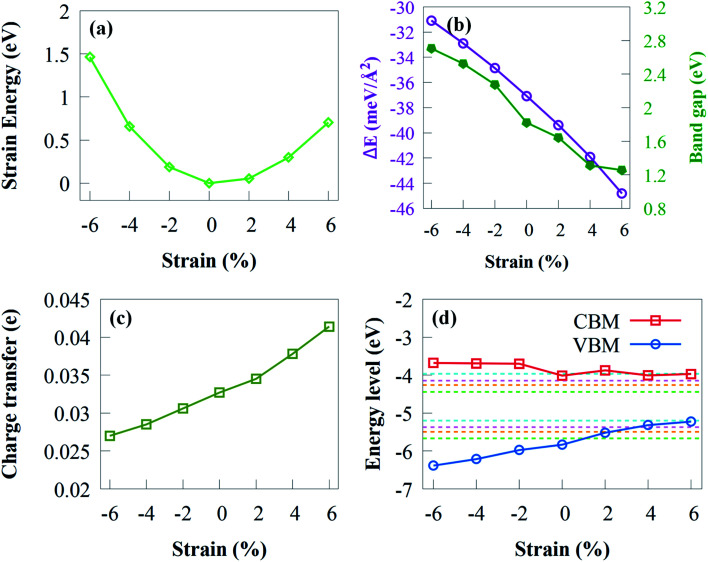
(a) Evolution of the strain energy of g-GaN/Sc_2_CO_2_ heterostructure with biaxial strain. Effect of strain on (b) binding energy, (c) bandgap, (d) charge transfer and (d) band edge position of g-GaN/Sc_2_CO_2_ heterostructure. The green, orange, magenta and cyan dashed lines represent the redox potentials at pH of 0, 3, 5 and 8, respectively, for water splitting.

The strain energy (*E*_s_) under biaxial strain is first discussed before analysing the electronic properties as follows:*E*_S_ = *E*_T_(*ε*_1_) − *E*_T_(*ε*_0_),where *E*_T_(*ε*_1_) and *E*_T_(*ε*_0_) are the total energies of biaxial strain and unstrain, respectively. It is worth noting that the variation curve of *E*_S_ in Fig. 8a indicates that g-GaN/Sc_2_CO_2_ heterostructure is flexible and still maintains the crystal structures of Sc_2_CO_2_ and g-GaN monolayers in the heterostructure under strain from −6 to +6%.

It is noteworthy that the *E*_b_ in Fig. 8b is always negative, implying the stability of g-GaN/Sc_2_CO_2_ vdW heterostructure under strain of −6 to +6%. The *E*_b_ decreases rapidly when biaxial strain changes from −2 to −6%. Nevertheless, the *E*_b_ under tensile strain increases monotonically when tensile strain increase from +2 to +6%. It is worth noting that *E*_b_ is less/more negative with increasing compressive/tensile strain, which indicates that enhanced stability is achieved when tensile biaxial strains are applied.

The curve regarding the bandgap under strain shows a linear variation. As shown from Fig. 8b, the bandgap decreased monotonically with tensile biaxial strain changing from +2 to +6%. The bandgap drops rapidly, indicating that high tensile biaxial strain may not favour water splitting process when g-GaN/Sc_2_CO_2_ is used as a photocatalyst. However, when the compressive biaxial strain varies from −2% to −6%, the bandgap monotonically increases. By applying biaxial strain, the bandgap energies exhibit very sharp change, which indicates that the electronic properties are sensitive to compressive or tensile strain.

The charge transfer from g-GaN monolayer to Sc_2_CO_2_ monolayer of various biaxial strained g-GaN/Sc_2_CO_2_ heterostructure is shown in Fig. 8c. The electron transfer reaches its maximum and minimum value at biaxial strain of +8% and −8%, respectively. The amount of transferred electrons increased as the strength of tensile strain increases. The enhanced charge transfer implies that the interaction between the g-GaN and Sc_2_CO_2_ monolayers is stronger compared with compressive strain, which results in the reduced bandgap energy.

We have also estimated the band edge positions of g-GaN/Sc_2_CO_2_ heterostructure under biaxial strain (Fig. 8d). When tensile strain is applied, the VBM is lower than the oxidation potential, which indicates that the heterostructure is not suitable for generating O_2_ but can serve as a potential photocatalyst to generate H_2_; thus, the reduction potential is satisfied. As a result of tensile strain, the VBO decreases, lowering the driving power for the holes moving from Sc_2_CO_2_ to g-GaN. This means that the overall photocatalytic water splitting cannot be achieved when tensile biaxial strain varies from +2% to +6%. In contrast, when compressive biaxial strain varies from −2% to −6%, the VBM and CBM gradually move away from the oxidation and reduction potentials, respectively. This result indicates that compressive strain can improve the photocatalytic efficiency of g-GaN/Sc_2_CO_2_ vdW heterostructure to some extent.

## IV. Conclusions

In summary, we have systematically investigated the interfacial interactions, charge density difference, band alignment, work function, electronic, structural and photocatalytic properties of novel 2D g-GaN/Sc_2_CO_2_ heterostructure through first-principles DFT calculations. The significant role of external electric field and biaxial strain on the structural and electronic properties were investigated in detail. The results showed that g-GaN and Sc_2_CO_2_ were in contact and formed a stable GaN/Sc_2_CO_2_ heterostructure. Using phonon dispersion and AIMD simulations, the thermal and dynamic stability of g-GaN/Sc_2_CO_2_ heterostructure was confirmed and easy to synthesise in experiments. g-GaN/Sc_2_CO_2_ heterostructure exhibits an indirect bandgap semiconductor with an inherent type-II band alignment, which can effectively separate and transfer charge carriers, thereby enhancing the photocatalytic activity. Besides, a large built-in electric field was found at the heterostructure interface, which will enhance the lifetime of charge carriers. Both charge density difference and electrostatic potential reveal electrons transferring from g-GaN monolayer to Sc_2_CO_2_ monolayer. Compared with the monolayers, g-GaN/Sc_2_CO_2_ vdW heterostructure possessed improved visible-light optical absorption intensity, ensuring the high-efficiency photocatalytic activity. The band alignment reveals that g-GaN/Sc_2_CO_2_ vdW heterostructure achieve overall water splitting at pH 0 to 7. Changing the strength and direction of external electric field and biaxial strain, the bandgap energy and band alignment of g-GaN/Sc_2_CO_2_ heterostructure could be effectively tuned in a broader range. Compressive strain and negative electric field were found to retain the reduction capability of Sc_2_CO_2_ and the oxidation capability of g-GaN, and this could significantly promote the hydrogen production from the overall water splitting. Most importantly, because of the influence of applied electric field and biaxial strain, g-GaN/Sc_2_CO_2_ heterostructure offer fundamental insights into the design of efficient photocatalyst with potential applications in solar energy conversion, high-performance nanoelectronics and optoelectronics devices.

## Conflicts of interest

The authors declare no conflicts of interest.

## Supplementary Material
